# Mechanism of pH-sensitive Amphiphilic Endosomal Escape of Ionizable Lipid Nanoparticles for Cytosolic Nucleic Acid Delivery

**DOI:** 10.1007/s11095-025-03890-8

**Published:** 2025-07-08

**Authors:** Zheng-Rong Lu, Da Sun

**Affiliations:** Department of Biomedical Engineering, Case Western Reserve University, Wickenden Building, Room 427, 10900 Euclid Avenue, Cleveland, OH 44106-7207 USA

**Keywords:** endosomal escape, ionizable lipids, LNP, nucleic acid delivery, pH-sensitive cell membrane disruption

## Abstract

Lipid nanoparticles (LNPs) are among the most successful classes of nonviral delivery systems for nucleic acid-based therapeutics in treating human diseases. One of the key challenges in achieving efficient cytosolic delivery of nucleic acids is overcoming endosomal entrapment within cells. Conventional lipid bilayer-forming cationic and amino lipids mediate endosomal escape via the mechanism of lamellar-to-inverted hexagonal phase transition, resulting in suboptimal cytosolic cargo delivery. pH-sensitive amphiphilic cell membrane disruption and endosomal escape have emerged as a strategy for designing protonatable or ionizable lipids, especially nonlamellar lipids, for efficient cytosolic nucleic acid delivery. Nonlamellar amino lipids possess a large wedge-shaped tail structure and do not form stable lipid bilayers. These lipids and their corresponding LNPs remain neutral, non-amphiphilic, or minimally amphiphilic at physiological pH (7.4). They become amphiphilic upon protonation or ionization in acidic endosomes (pH 6.5–5.4). The electrostatic interaction of ionized nonlamellar lipids with the negatively charged endosome membrane, combined with their large wedge-like structures, disrupts the lipid bilayer, facilitating efficient endosomal escape. Additionally, the nonlamellar ionizable lipids can be fine-tuned by altering the structure of amino head groups and lipid tails to achieve the precisely controlled pH-sensitive amphiphilic membrane disruption at endosomal pH. Therefore, these lipids exhibit excellent safety profiles and high efficiency for *in vivo* delivery of various therapeutic nucleic acids. pH-sensitive amphiphilic membrane disruption and endosomal escape provide a feasible and effective mechanism for designing ionizable lipids for safe and efficient *in vivo* nucleic acid delivery.

## Introduction

Significant progress has been made in the clinical application of nucleic acid therapeutics. Various nucleic acids, including DNA, siRNA, miRNA, mRNA, lncRNA, and CRISPR/Cas, have been explored as a therapeutic approach to edit, regulate, or replace target genes, and to serve as vaccines to prevent and treat human diseases. The clinical application of nucleic acid therapeutics requires safe and efficient cytosolic delivery into target cells. Since the inception of gene therapy, the design and development of delivery systems for nucleic acids have been a critical focus [1]. Lipid-based delivery systems have emerged as the most successful class of nonviral systems for nucleic acids. Various nucleic-acid loaded lipid nanoparticles (LNPs) have been approved for clinical applications following decades of continuous innovation by multiple generations of scientists [2–14]. Recently, substantial progress has been made in the development and clinical translation of pH-sensitive protonatable or ionizable amino lipids for efficient cytosolic delivery of nucleic acids. Numerous ionizable lipids have been developed and tested for nucleic acid delivery [6, 11, 15–21], and some exhibit significant pH-sensitive destabilization of cell and subcellular membranes, enabling safe and efficient cytosolic delivery [19, 22–26]. The success of ionizable LNPs has been exemplified by the mRNA vaccines used to combat the COVID-19 pandemic, which have saved millions of lives globally [27, 28]. Despite the success, there remains a critical need for safer and more effective delivery systems to enable broader clinical applications of various nucleic acid therapeutics [12, 23, 29–31].

One of the main considerations in designing efficient nucleic acid delivery systems is ensuring their escape from endosomes following endocytosis [32]. Theories such as the “proton sponge effect” and the lamellar-to-inverted hexagonal phase transition are commonly applied to guide the design of cationic polymer and lipid-based delivery systems, respectively [33–39]. Liposomes represent the first generation of LNPs for nucleic acid delivery. Various pH-sensitive lipids, amphiphiles, and surfactants, including oleic acid, phosphatidylethanolamine (PE), and dioleoylphosphatidylethanolamine (DOPE), have been incorporated into lipid bilayers to develop pH-sensitive liposomes [1, 2, 4, 40, 41]. The ionic state of these lipids changes in response to the acidic pH of endosomes, inducing a lamellar-to-inverted hexagonal phase transition. This phase transition promotes fusion with cytoplasmic membranes, destabilizing the lipid bilayer, facilitating endosomal escape, and enabling cytosolic delivery of nucleic acids [34, 42]. The lamellar-to-inverted hexagonal phase transition has been widely used to explain the mechanism of endosomal escape in pH-sensitive liposomes, as well as in a few ionizable lipids [12, 23, 29–31, 43]. However, the efficiency of cytosolic nucleic acid delivery via pH-sensitive liposomes that rely on this mechanism remains limited. Nevertheless, this mechanism has been extensively reported in literature [12, 23, 29–31, 39, 43] and will not be the focus of this review.

**Fig. 1:**
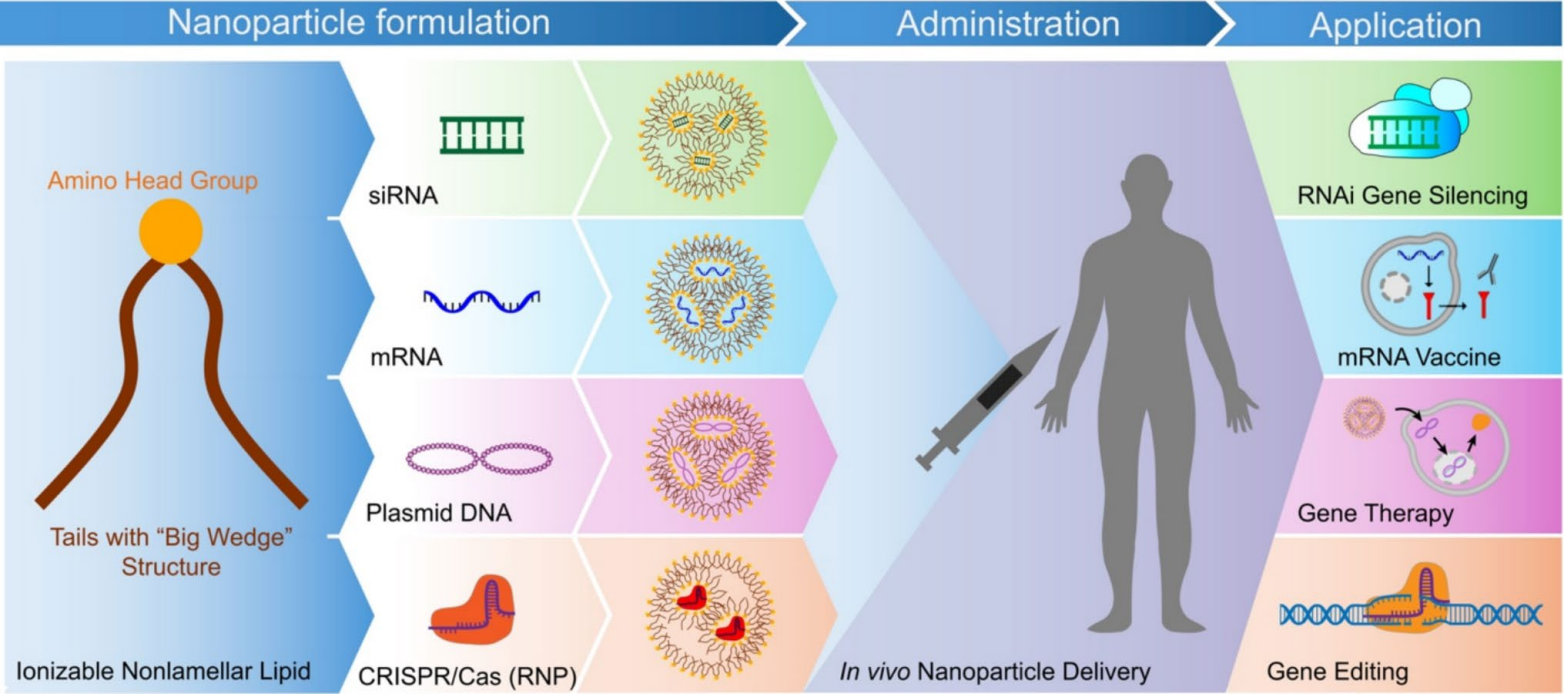
Nonlamellar ionizable lipid nanoparticles facilitate safe and efficient cytosolic delivery of a variety of therapeutic nucleic acids, including small RNA, mRNA, plasmid DNA, and gene editors into target cells.

Here, we discuss the mechanism of pH-sensitive amphiphilic membrane disruption and endosomal escape in the design of highly efficient ionizable lipids for safe and effective cytosolic delivery of nucleic acid therapeutics. We hypothesize that precisely controlling the pH-sensitive amphiphilicity of nonlamellar amino lipids can minimize nonspecific *in vivo* tissue interactions of their LNPs, enhancing safety during systemic delivery while facilitating efficient endosomal escape and cytosolic delivery after endocytosis in target cells [19, 44, 45]. Nonlamellar ionizable lipids, which do not form stable lipid bilayers, can achieve precisely controlled pH-sensitive amphiphilicity, exhibiting no or minimal cell membrane disruption at neutral pH while promoting efficient endosome membrane destabilization at acidic pH, and enhanced cytosolic delivery efficiency [19, 20, 45]. These lipids demonstrate excellent safety and efficacy for *in vivo* delivery of therapeutic nucleic acids of any size (Fig. 1) [46–49]. We illustrate the concept of pH-sensitive amphiphilic membrane disruption and endosomal escape, and highlight the potential of nonlamellar ionizable lipids with precisely controlled pH-sensitive amphiphilicity for efficient cytosolic nucleic acid delivery.

## The Mechanism of pH-sensitive Amphiphilic Endosomal Escape

The cell and subcellular membranes consist of a lipid bilayer containing phospholipids, cholesterol, and transmembrane proteins, playing a critical role in regulating the transport of substances into and out of cells and subcellular compartments. Nucleic acids, being negatively charged macromolecules, cannot pass through cell membranes unaided. Amphiphiles or surfactants can interact with the lipid bilayer, disrupting the membrane to facilitate the delivery process. Cationic lipids, such as DOTAP (1,2-dioleoyl-3-trimethylammonium propane), are positively charged amphiphiles that have been incorporated into cationic liposomes for nucleic acid delivery [50]. These cationic liposomes form stable LNPs with negatively charged nucleic acids, enabling intracellular transfection. However, these cationic lipids generally exhibit relatively low transfection efficiency and significant toxicity.

Ionizable lipids demonstrate promise in overcoming the limitations of cationic lipids, offering a more effective and safer approach for *in vivo* cytosolic delivery of nucleic acids. These lipids mitigate cytotoxicity concerns, providing a pathway for efficient nucleic acid delivery in therapeutic applications [3, 5, 6, 8, 9, 40, 51]. As early as the 1980 s, it was reported that incorporating ionizable amino lipids into liposomes could induce pH-responsive destabilization of lipid bilayers [34, 41, 42, 52–54]. This effect is attributed to changes in the ionization state of the lipids at acidic pH, triggering a lamellar-to-inverted hexagonal phase transition that destabilizes the liposomes [34, 55]. However, direct evidence supporting the ability of lamellar ionizable lipids, which form stable lipid bilayers and readily incorporate into liposomes to promote efficient endosomal escape, remains limited. For instance, LNPs formulated with lamellar ionizable lipids such as DODAP, Dlin-KC2-DMA (KC2), and DLin-MC3-DMA (MC3) were developed to facilitate effective endocytosis [8, 10, 56–58]. Although some reports suggest that these LNPs mediate cytosolic delivery, findings regarding their ability to induce endosomal escape remain mixed. One study showed that LNPs containing KC2 enabled the dispersed distribution of siRNA-Cy5 in the cytoplasm of bone marrow macrophages and dendritic cells [10]. However, other studies failed to reliably detect endosomal escape mediated by KC2 or MC3 [24, 59, 60]. These findings suggest that the formation of a stable lamellar structure in ionizable lipids may restrict lipid mobility, slowing the pH-responsive phase transition [61, 62]. Consequently, the lamellar-to-inverted hexagonal phase transition may act as a rate-limiting step for the endosomal escape of LNPs composed of lamellar ionizable lipids.

In 2007, we hypothesized that nonlamellar protonatable or ionizable amino lipids with precisely controlled pH-sensitive cell membrane disruption at endosomal pH (6.5–5.4) could facilitate efficient amphiphilic endosomal escape and cytosolic nucleic acid delivery [19]. Unlike lamellar lipids, nonlamellar amino lipids do not form lipid bilayers, thereby bypassing the rate-limiting lamellar-to-inverted hexagonal phase transition and enabling rapid endosomal escape. Nonlamellar amino lipids readily self-assemble into stable lipid nanoparticles (LNPs) capable of encapsulating nucleic acids of any size. At physiological neutral pH, these amino lipids and their LNPs remain neutral and non-amphiphilic, or weakly charged with minimal amphiphilicity, exhibiting little to no cell membrane disruption or cytotoxicity during systemic circulation. Following endocytosis, the amino lipids in the LNPs become protonated, transitioning to an amphiphilic state in the early endosome (pH ~ 6.5) and late endosome (pH ~ 5.4). This protonation induces electrostatic and amphiphilic interactions with the endosomal membrane, destabilizing it and enabling effective endosomal escape and cytosolic release of nucleic acids [19, 44].

**Fig. 2:**
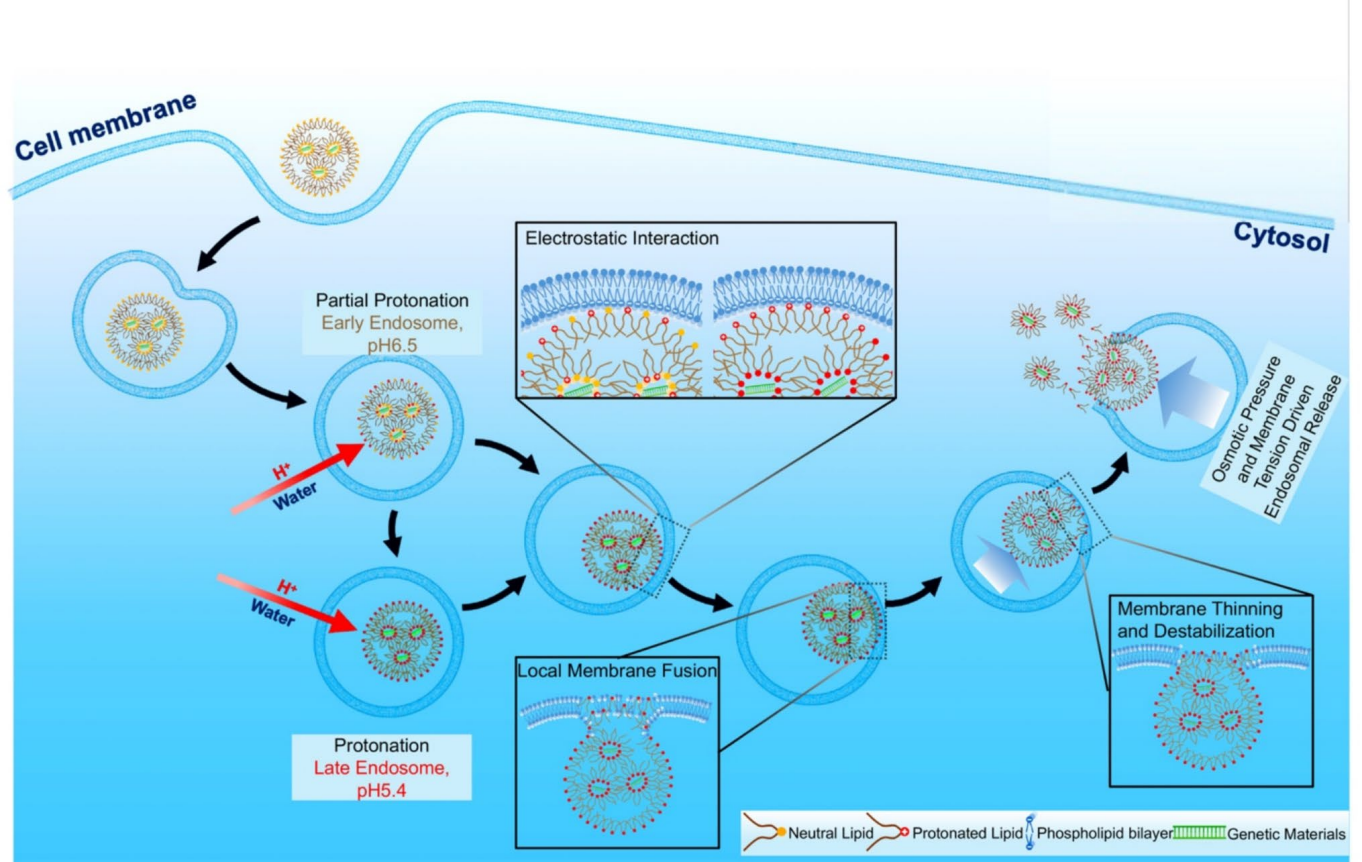
Illustration of the mechanism of pH-sensitive amphiphilic membrane disruption and endosomal escape of nonlamellar ionizable lipid nanoparticles after endocytosis. The process involves 1) partial or strong protonation of the ionizable lipids in the LNP in acidic early or late endosome; 2) electrostatic interactions of the cationic lipids of the LNP with the negatively charged membrane; 3) local fusion of the cationic nonlamellar lipids within the endosomal membrane; 4) local disruption, thinning, and consequent destabilization of the lipid bilayers by the fused nonlamellar lipids with their large wedge lipid structure; 5) escape of the LNP from the endosome at the disruption site, driven by increased osmotic pressure and endosomal membrane tension induced by the influx of protons and water due to the protonation of the ionizable lipids.

As illustrated in Fig. 2, the pH-sensitive amphiphilic endosomal escape process may involve the following steps: 1) partial to strong protonation of the nonlamellar ionizable lipids in the LNP within early or late endosomes following endocytosis; 2) electrostatic interactions between the cationic head groups of the ionized lipids in the LNP and the negatively charged endosomal membrane; 3) local fusion of the ionized nonlamellar lipid molecules within endosomal membrane; 4) local disruption and thinning of the lipid bilayers induced by their large wedge-like structure, leading to the destabilization of the membrane; 5) escape of the LNP from the endosome at the site of membrane destabilization driven by increased osmotic pressure and membrane tension due to their protonation and the consequent influx of protons and water. This controlled process occurs locally at the contact point between the LNP and the endosomal membrane, preventing significant membrane damage and minimizing potential adverse effects. This specificity ensures the safe and efficient cytosolic delivery of nucleic acids.

## Structural Effects on pH-sensitive Amphiphilic Membrane Desruption of Ionizable Lipids

The structure of protonatable or ionizable lipids plays a crucial role in controlling their pH-sensitive cell membrane disruption. Ionizable lipids that form stable lamellar structures often exhibit suboptimal cytosolic nucleic acid delivery because their stable lamellar arrangement restricts pH-sensitive phase transitions and limits amphiphilic interactions with the endosomal membrane [59, 60]. To address this limitation, we incorporated these structural features in ionizable lipids: 1) amino lipids with a large wedge structure that do not form a stable bilayer, 2) non-amphiphilicity or low amphiphilicity of the nonlamellar amine lipids and their lipid nanoparticles (LNPs) at neutral pH for safe systemic delivery, 3) protonation or ionization of the nonlamellar amino lipids and their LNPs enabling amphiphilic lipid bilayer membrane disruption precisely at endosomal pH. The nonlamellar ionizable lipids with the features are capable of precise pH-sensitive amphiphilic membrane disruption at endosomal pH [19, 20, 63, 64]. This design strategy ensures minimal or no amphiphilicity at neutral pH, with a controlled transition to amphiphilic cationic lipids that can destabilize endosomal membranes in an acidic environment.

**Fig. 3:**
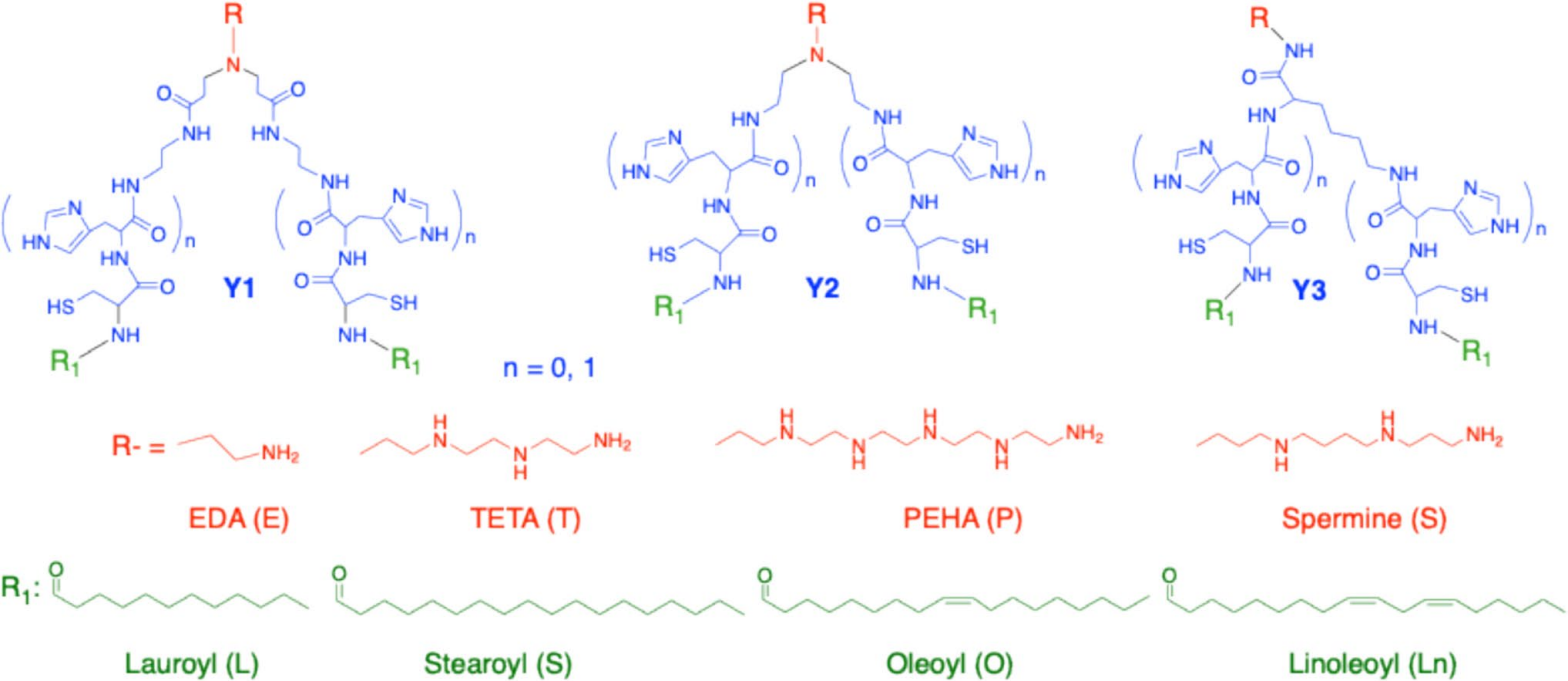
The general structures of nonlamellar pH-sensitive amphiphilic amino lipids with different hydrophilic “Y” joints.

We have introduced distant lipid tails to form a large wedge-shaped structure in amino lipids to design nonlamellar ionizable lipids. Three hydrophilic “Y” joints have been utilized to increase the distance between the dual lipid tails, ensuring a large wedge-shaped conformation in the amino lipids [19, 20, 63, 64]. The general structures of the designed lipids are illustrated in Fig. 3. These “Y” joints have 13—27 atoms to separate the lipid tails, and effectively prevent the formation of stable lipid bilayers, enabling efficient and controlled pH-sensitive membrane destabilization.

The amino head groups and lipid tails were systematically varied to fine-tune the overall structural features of the ionizable lipids and optimize their pH-sensitive amphiphilicity. The amino head groups, including ethylenediamine (EDA, E), triethylenetetraamine (TETA, T), pentaethylenehexamine (PEHA, P), spermine (S), and histidine (H), were tested to evaluate the effects of protonatable nitrogen density and cationic charges on pH-sensitive amphiphilicity. These head groups were combined with various lipid tails, such as lauroyl (L), oleoyl (O), linoleoyl (Ln), and stearoyl (St) to assess their influence on pH-responsive interactions with cell membranes. Additionally, two cysteinyl (C) residues were incorporated to introduce polymerizable thiol groups, which can stabilize lipid nanoparticles (LNPs) via redox-sensitive disulfide bond formation and enable surface modifications (Fig. 3). Notably, these multifunctional pH-sensitive amino lipids do not form stable liposomes under conventional liposome-forming conditions with other helper lipids. Instead, they spontaneously self-assemble into stable LNPs with siRNA, miRNA, mRNA, and plasmid DNA without the need for additional helper lipids [19, 20, 46, 63–67].

**Fig. 4:**
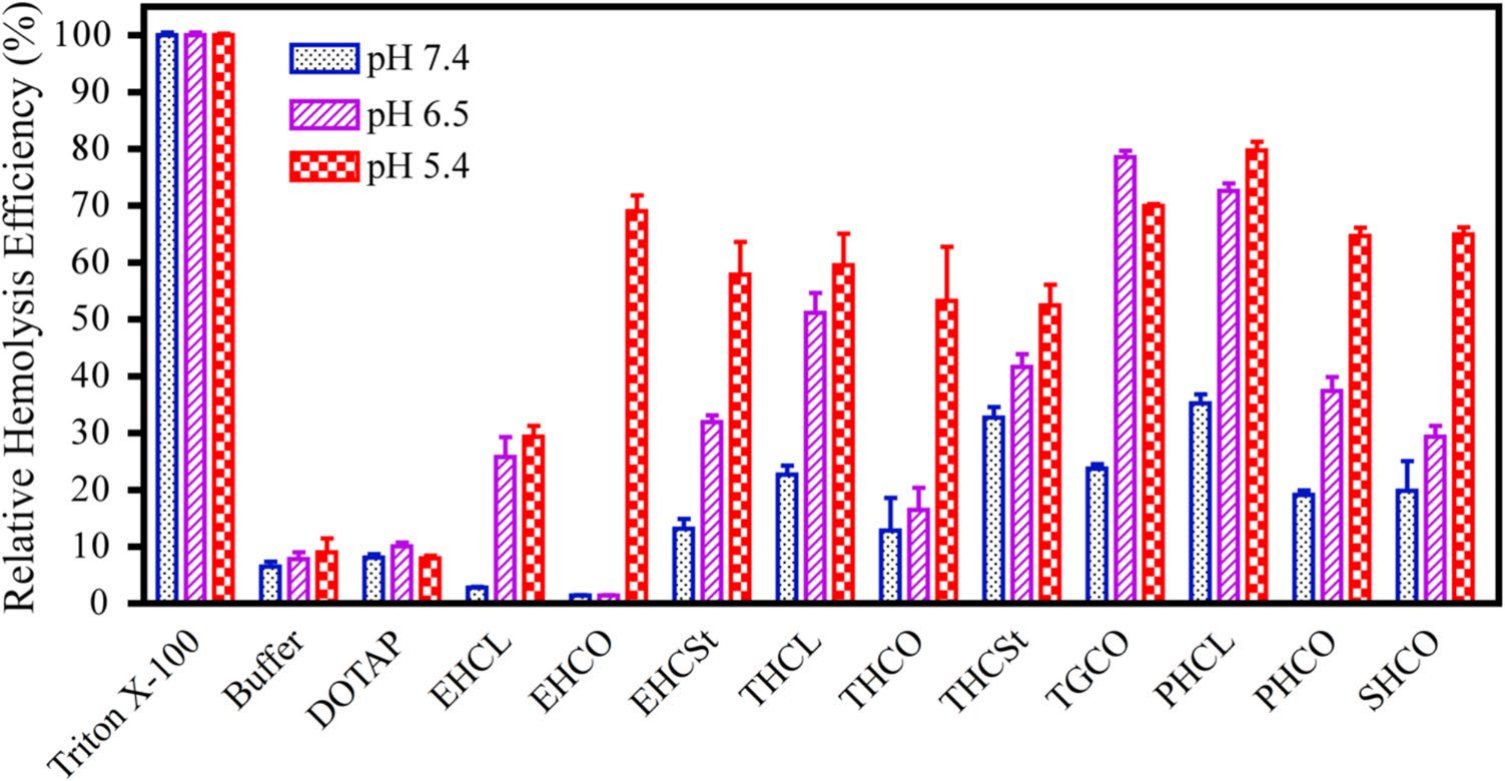
pH-sensitive hemolytic activity or amphiphilic cell membrane disruption of the multifunctional amino lipids with “Y1” joint from Fig. 3 [19]. E, T, P, and S denote ethylenediamine (E), triethylenetetramine (T), pentaethylenehexamine (P), and spermine (S) in the amino head groups; H, C, and G for histidine, cysteine, and glycine in the Y joints; L, O, and St for lauroyl, oleoyl, and stearoyl chains.

The pH-sensitive, amphiphilic cell membrane destabilization of these lipids and their LNPs was assessed by measuring their hemolytic activity at physiologically neutral pH (7.4), early endosomal pH (6.5), and late endosomal pH (5.4). The number of ionizable amines in the head group, the structure of the lipid tails, and the distance between the lipid tails all play critical roles in pH-sensitive amphiphilic cell membrane disruption, as reflected by the hemolytic activity. The membrane-disruptive activity of most amino lipids increased as pH decreased to 6.5 and 5.4 (Fig. 4). The lipids with more than 2 amino groups exhibited substantial membrane destabilization at neutral pH, possibly due to substantial protonation and high charge density of the protonation. The presence of histidyl residues resulted in less membrane destabilization at pH 7.4 and 6.5 compared to lipids without histidine, possibly due to the strong buffering capacity of the imidazole groups (pK_a_ ≈ 6.0). Unsaturated lipids demonstrated greater pH-sensitive cell membrane disruption than their saturated counterparts [19]. The LNPs of the ionizable lipids followed the same trend. However, no significant difference was observed between oleoyl and linoleoyl tails in amino lipids with the same head groups [20]. In general, the lipids with a head group containing two or less ionizable amines, excluding imidazole group in histidine, and unsaturated lipid tails exhibited precisely controlled pH-sensitive hemolytic activity with little membrane disruption at pH 7.4, and increased disruption activity when pH decreases from pH = 6.5 to 5.4 [19, 20, 63]. The lack of cell membrane disruption at neutral pH is critical for improving hemocompatibility and minimizing cytotoxicity of the ionizable LNPs for safe systemic delivery of nucleic acids.

Interestingly, the control cationic lipid DOTAP, which contains a small “Y” joint (only four atoms between the lipid tails) and has permanent amphiphilicity, did not exhibit significant cell membrane disruption at the tested pH values (Fig. 4) [19, 63]. Similarly, the plasmid DNA LNP of the commercial transfection agent Lipofectamine 2000, which contains a 3:1 mixture of the ionizable lipids DOSPA (2,3‐dioleoyloxy‐N‐[2(sperminecarboxamido)ethyl]‐N,N‐dimethyl‐1‐propaniminium trifluoroacetate, also with four atoms between the lipid tails) and DOPE exhibited only a slight increase in cell membrane destabilization at pH 5.5 compared to pH 7.4 [67]. These lipids with small “Y” joints are likely to form stable lamellar structures, resulting in limited cell membrane destabilization [19, 63, 67]. Similarly, LNPs containing the ionizable lipids KC2 and MC3 have been reported to possess lamellar characteristics and did not show strong pH-sensitive hemolytic activity (~ 12% or less), even at pH 5.4 [24, 62]. These findings indicate that the distance between lipid tails plays a critical role in pH-sensitive cell membrane disruption. Ionizable lipids with two or fewer protonatable amines and distant unsaturated lipid tails exhibit preferable and precisely controlled amphiphilic membrane destabilization at endosomal pH, making them ideal for efficient endosomal escape and cytosolic delivery.

**Fig. 5:**
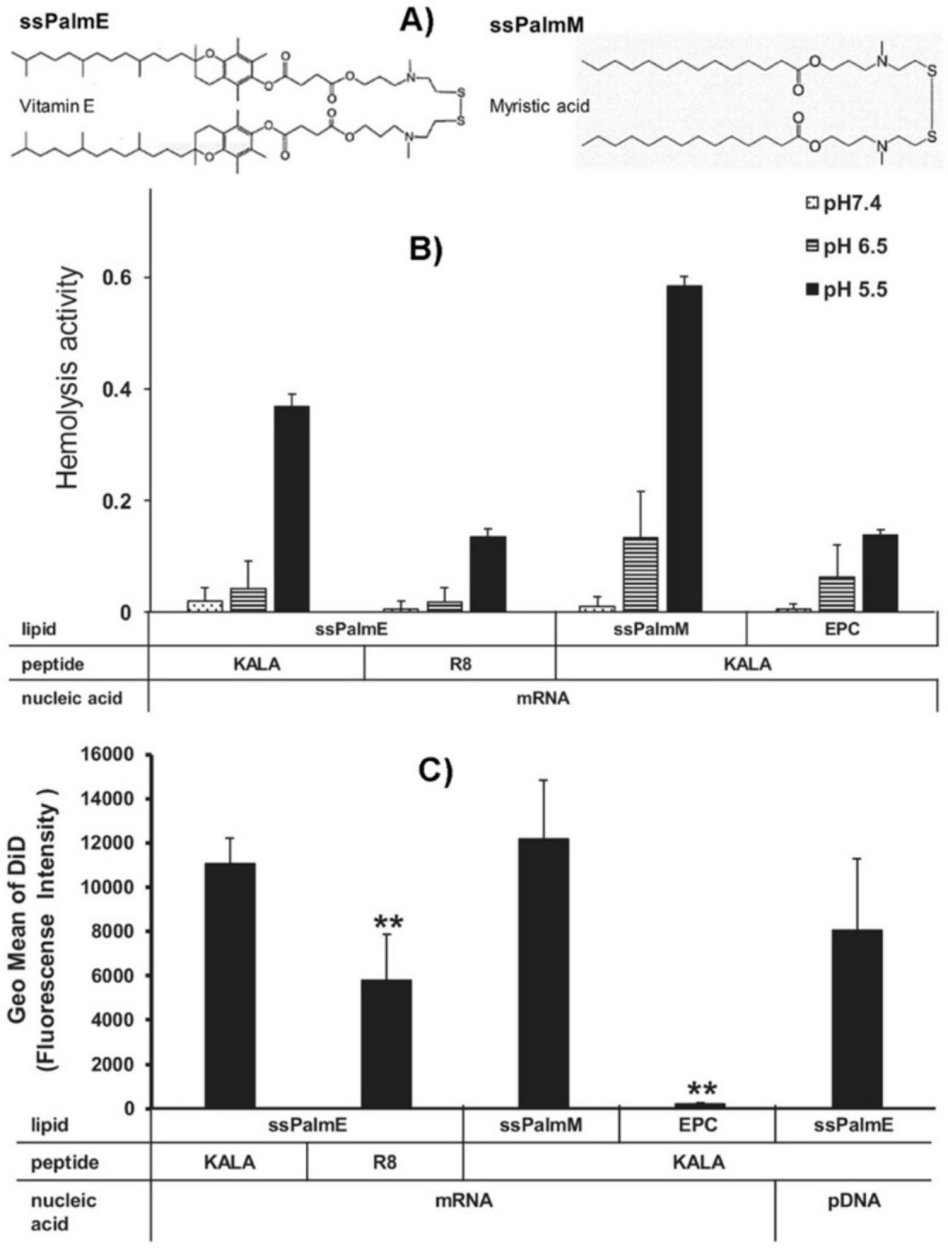
The LNPs containing nonlamellar lipids ssPalmE and ssPalmM (**A**) showed more effective pH-sensitive hemolysis (**B**) and higher cellular uptake (**C**) than the lamellar lipid EPC [68].

Recently, other types of ionizable lipids with large wedge-shaped structures, including those with distant lipid tails, multiple tails, and branched tails have been designed to overcome the limitations of lamellar ionizable lipids and enhance cytosolic nucleic acid delivery efficiency [15, 22, 25, 62, 68–71]. pH-dependent hemolytic assays have also demonstrated that the LNPs of some of these ionizable lipids exhibit effective pH-sensitive membrane disruption [22, 68]. For example, LNPs of the ionizable lipids ssPalmE and ssPalmM, which contain distant lipid tails, exhibited low hemolytic activity at neutral pH but high activity at late endosomal pH, leading to more efficient intracellular mRNA delivery than the lamellar lipid EPC (1,2-dilauroyl-sn-glycero-3-ethylphosphocholine chloride) (Fig. 5) [68]. These lipids adopt a nonlamellar structural morphology and have a strong tendency to induce pH-sensitive destabilization of lipid bilayers, particularly at endosomal pH.

**Fig. 6:**
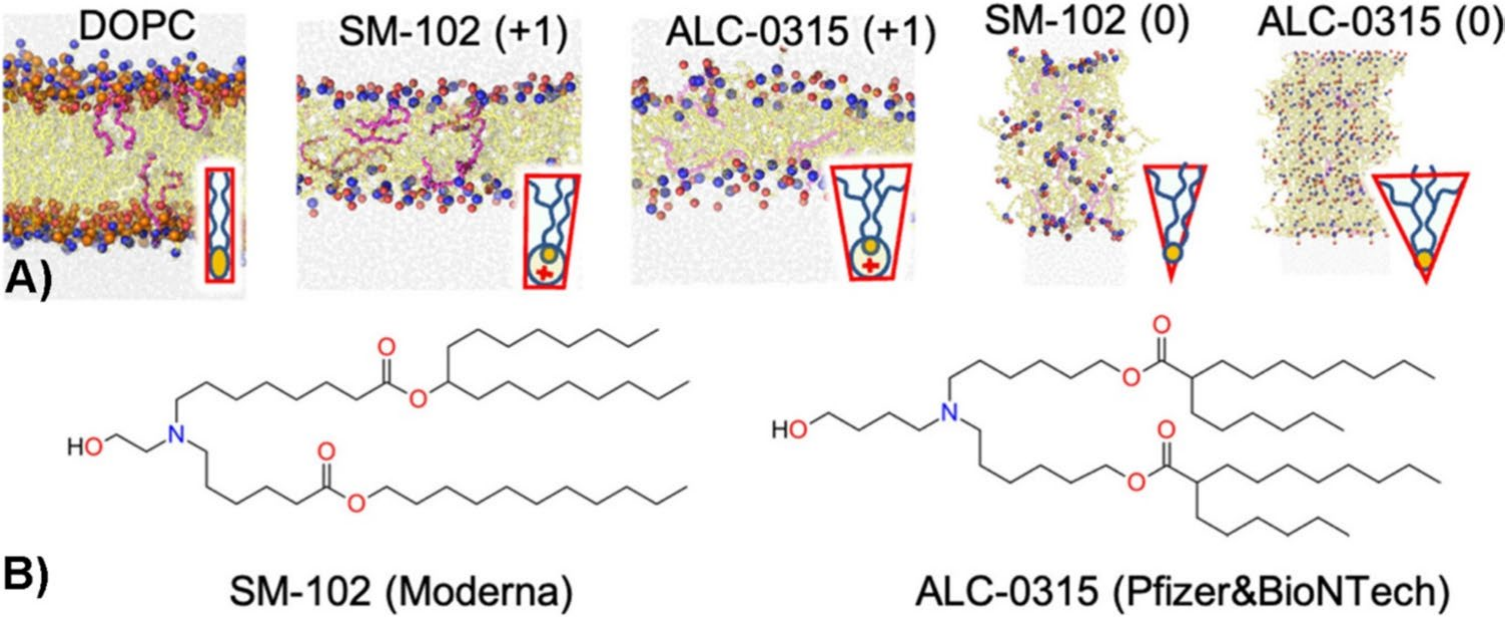
(**A**) Molecular dynamics simulations show final snapshots of pure ionized ALC-0315, SM-102, and DOPC bilayers with schematic depictions of the corresponding molecular structures in the insets. The neutral ALC-0315 and SM-102 do not form stable bilayer. (**B**) Structures of the ionizable lipids of SM-102 and ALC-0315*.*[72].

Molecular dynamics simulations have shown that the neutral forms of pure branched ALC-0315 and SM-102 lipids, which have a large wedge-shaped lipid structure, do not form stable lipid bilayers [72]. The ionized forms of ALC-0315 and SM-102 generate highly disordered and thinner bilayers compared to those formed with the lamellar lipid DOPC (1,2-dioleoyl-sn-glycero-3-phosphocholine) (Fig. 6) [72]. The thinning of bilayers containing the ionized lipids is attributed to the repulsion among the positively charged head groups and the large surface area occupied by their branched lipid chains. ALC-0315, which has quadruply branched tails, forms thinner bilayers (2.5 nm thick) than SM-102 (2.7 nm), which has triply branched tails, and DOPC, which has stable bilayers (3.8 nm) [72].

**Fig. 7:**
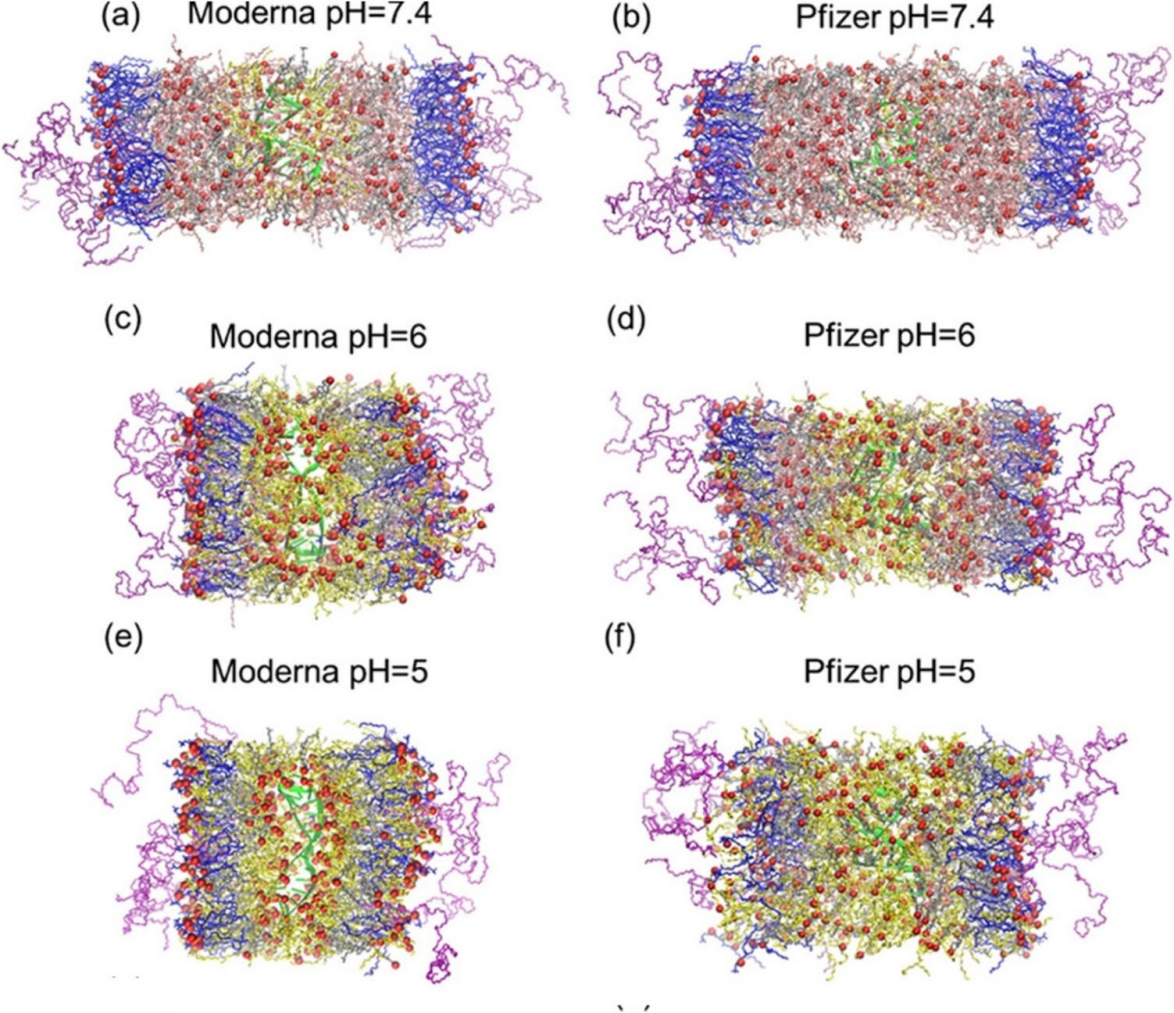
Molecular dynamics simulations of the mRNA LNPs of SM-102 (Moderna) and ALC-0315 (Pfizer) thinning of the lipid bilayers when pH decreases from 7.4 to 6 and 5 (a-f). Protonated lipids are shown in yellow, deprotonated ionizable lipids are shown in pink, DSPC is shown in blue, cholesterol is shown in gray, PEGylated lipid is shown in purple, RNA is shown in green, sodium and chloride ions are shown in blue and cyan beads, and the oxygen atoms of cholesterol, the nitrogen atoms of ionizable lipids, and the phosphorus atoms of DSPC are represented by red beads.[73].

Molecular dynamics simulations of mRNA LNPs containing SM-102 (molar ratio: 50 SM-102/38.5 cholesterol/10 DSPC/1.5 PEG2000-DMG) and ALC-0315 (46.3 ALC-0315/42.7 cholesterol/9.4 DSPC/1.6 PEGylated lipid ALC-0159) have shown that the protonated lipids are located on the surface of the LNPs [73]. As the pH decreases, the thickness of the lipid bilayers decreases while their surface area increases (Fig. 7). These structural changes in the lipid bilayers are also attributed to cationic charge repulsion and the large surface area occupied by the lipid tails [73].

Taken together, nonlamellar ionizable lipids possess the capability for pH-sensitive cell membrane destabilization. Their structure can be fine-tuned to achieve precisely controlled membrane disruption at endosomal pH. Upon protonation in the acidic endosome, the positively charged nonlamellar lipids at the surface of LNPs interact with negatively charged membranes. Additionally, the large surface area of protonated nonlamellar ionizable lipids contributes to membrane disruption and thinning in acidic endosomes. The molecular dynamics simulations show that the thinning of lipid bilayers composed of nonlamellar ionizable lipids at acidic endosomal pH supports the theory that the pH-sensitive amphiphilic disruption of the lipid bilayer membrane facilitates efficient endosomal escape for cytosolic nucleic acid delivery.

## pH-sensitive Amphiphilic Endosomal Escape of Ionizable LNP

**Fig. 8:**
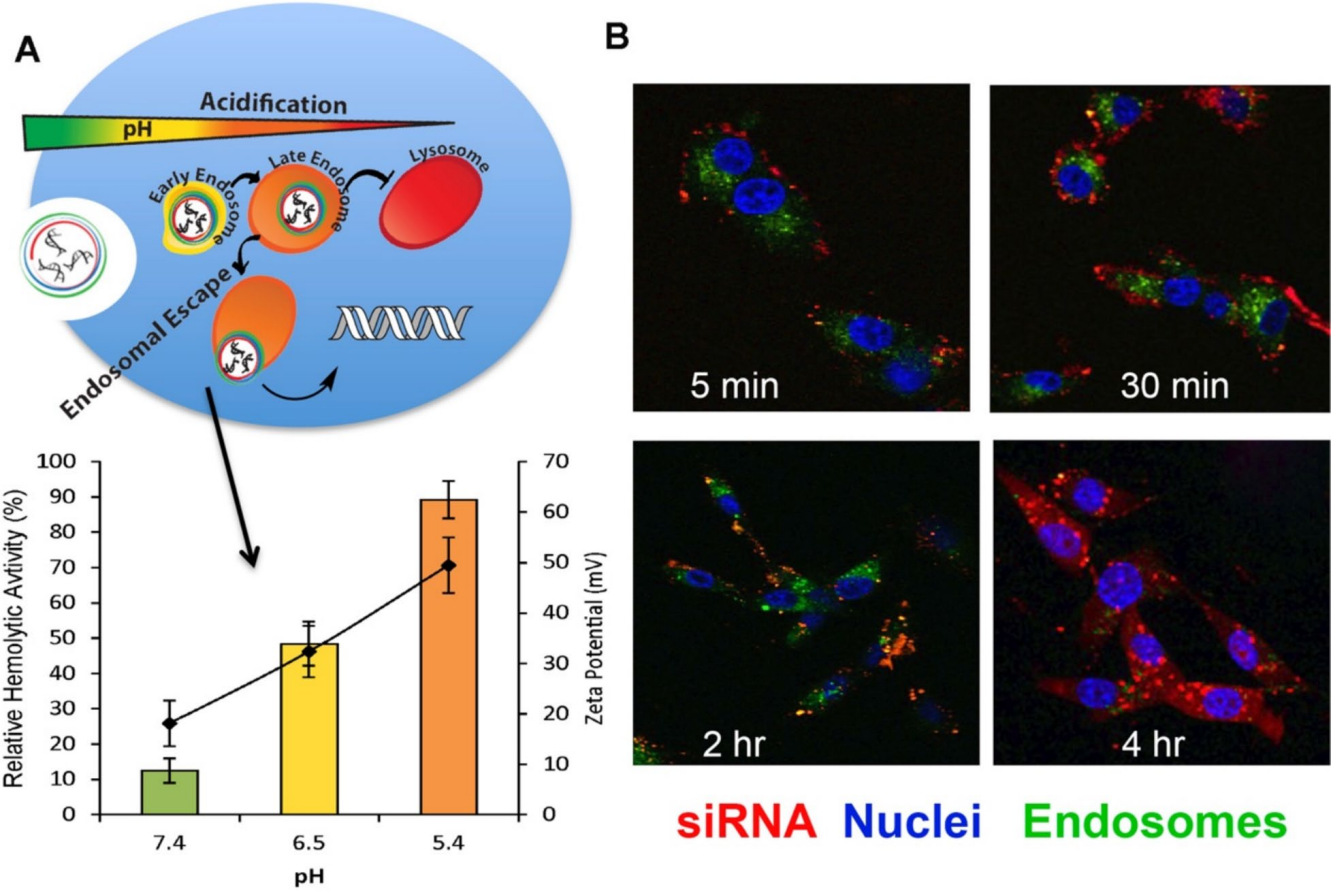
(**A**) Illustration of pH-sensitive endosome escape in correlation with pH-sensitive hemolytic activity and zeta potential (curve) of ECO/siRNA nanoparticles following incubation in PBS at various pH levels. The zeta potential was found to increase with increasing acidity. Hemolytic assay determined the pH-dependent membrane-disruptive ability of ECO/siRNA nanoparticles increased significantly (p < 0.05) with increasing acidity (pH = 7.4, 6.5, 5.4). Relative hemolytic activity calculated with respect to the hemolytic activity of 1% Triton X-100. (**B**) Immunofluorescence imaging using an LAMP1-antibody (Alexa Fluor 488-labelled secondary antibody) to stain for endosomes reveals colocalization (yellow color) of ECO/siRNA (Alexa Fluor 647-labelled siRNA) nanoparticles occurring as early as 5 min post-transfection. At 4 h, a dispersed siRNA signal is present within the cytosol, indicating that ECO/siRNA nanoparticles are able to escape from endosomes and release siRNA cargo in cytosol [44].

Nonlamellar multifunctional amino lipids containing ethylenediamine and unsaturated lipid tails, including ECO, EHCO, EKHCO, ECLn, EHCLn, and iECO exhibit excellent pH-sensitive cell membrane destabilization, robust nucleic acid transfection efficiency, and minimal cytotoxicity [19, 20, 63, 64]. These lipids self-assemble with negatively charged nucleic acids to form stable LNPs without the need for helper lipids [47, 74]. The nanoparticles are further stabilized through reducible disulfide crosslinks formed via autoxidation of thiols. ECO has been identified as the lead lipid for further investigation. ECO forms stable lipid nanoparticles (ELNPs) with siRNA [75, 76], miRNA [49, 66], plasmid DNA [46, 77], mRNA (unpublished), and ribonucleoprotein (unpublished) for efficient *in vitro* and *in vivo* nucleic delivery with an excellent safety profile. As pH decreases from 7.4 to 6.5 and further to 5.4, the protonation of the head group increases the zeta potential and cationic charge density of ELNPs. This protonation enhances the amphiphilicity of the lipid and its hemolytic activity, suggesting greater endosomal membrane disruption. Consequently, ELNPs efficiently escape from early and late endosomes, enabling rapid cytosolic delivery (Fig. 8A) [20, 44].

The cytosolic siRNA delivery capability of ELNP was first demonstrated using AF-488-labeled siRNA ELNP in HT29 colon cancer cells. Dispersion of the labeled siRNA was observed as early as 60 min post-transfection, indicating early endosomal escape and siRNA release into the cytosol [20]. Further investigation into ECO-mediated endosomal escape and cytosolic siRNA delivery was conducted through colocalization studies using AF-488-labeled anti-LAMP antibodies in U87 cells (Fig. 8B). Colocalization of AF-647-labeled siRNA ELNPs with AF-488-labeled endosomes was detected as early as 5 min post-transfection, with substantial colocalization observed at 2 h, suggesting subcellular trafficking within endosomes. By 4 h, significant cytosolic dispersion of AF-647-labeled siRNA was evident, indicating effective internalization of ELNPs, endosomal escape, and siRNA release into the cytosol [44].

**Fig. 9:**
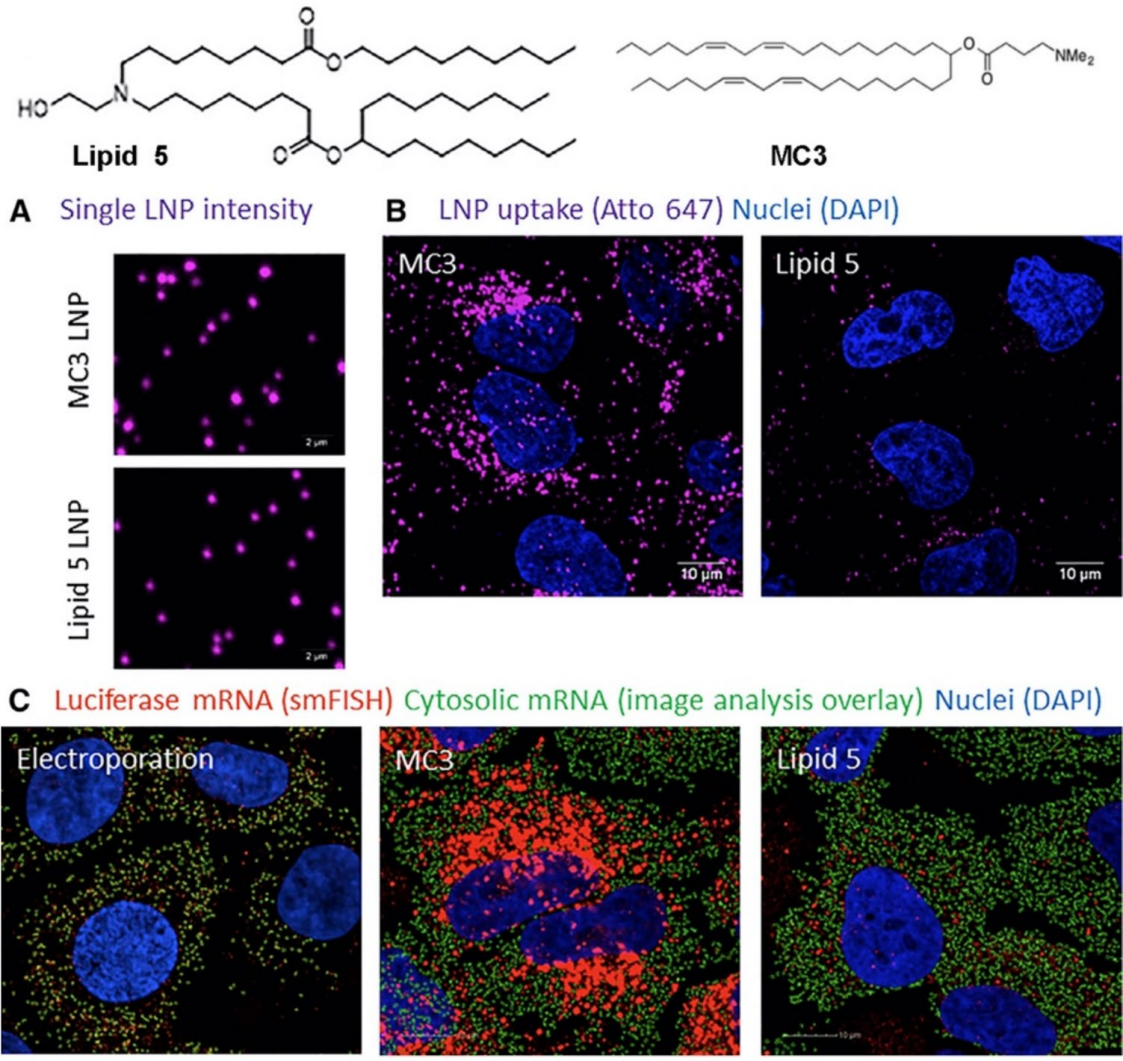
(**A**) Fluorescence images of ATTO 647-labeled MC3, and lipid 5 mRNA LNPs (magenta) on glass. (**B**) Fluorescence images showing the uptake of ATTO 647-labeled LNP 4 h after transfection in HeLa cells. (**C**) Cytosolic mRNA delivery by MC3 and lipid 5 as shown by Stellaris single-molecule fluorescence *in situ* hybridization (smFISH) processing [25].

The branched ionizable lipids also induce more efficient endosomal escape than lamellar lipid MC3 in mRNA LNPs [25, 69]. A study compared the cellular uptake and endosomal escape of MC3 with lipid 5, an analogue of SM-102, using single molecule fluorescence microscopy. MC3 LNP produced higher cellular uptake than Lipid 5, however, lipid 5 resulted in a 6-fold increase of endosomal escape efficiency as compared to MC3 LNP, about 15% and 2.5% cytosolic delivery efficiency for Lipid 5 and MC3, respectively, Fig. 9 [25]. Parallel-tempered metadynamics simulations confirmed that the branched ionizable lipids SM-102 and ALC-0315 possessed low free energy value and high capacity to penetrate a lipid bilayer, while MC3 did not have the ability to pass through a bilayer [78]. Consequently, the branched ionizable lipids were more likely to mediate mRNA transfection for vaccines [69]. The results further demonstrate that nonlamellar ionizable lipids are more effective in inducing endosomal escape than lamellar ionizable lipids.

Biocompatibility and hemocompatibility of the delivery systems are essential for nucleic acid therapeutics because they are mostly administered systemically or locally. The pH-sensitive nonlamellar ionizable lipids and LNPs take advantage of the difference between physiological and endosomal pH levels to precisely control their electrostatic and amphiphilic interactions with cell and subcellular membranes. The absence of cell membrane disruption of the ionizable LNPs at the neutral pH improves their biocompatibility and hemocompatibility for safe systemic and local delivery. The mRNA LNPs of SM-102 and ALC-0315 have been used as vaccine to combat COVID-19 pandemic. ELNPs have shown excellent safety and efficacy for systemic and local delivery of nucleic acids of various sizes, including miRNA, siRNA, and plasmid DNA for cancer therapy and gene therapy of retinal genetic disorders [46, 49, 66, 75–77, 79]. The ELNP of CRISPR/Cas plasmids also produced high expression of sgRNAs and Cas9 protein and gene silencing efficacy *in vitro* [64].

### Summary

Endosomal escape of ionizable lipid nanoparticles (LNPs) is one of the most critical steps in the effective cytosolic delivery of therapeutic nucleic acids. The concept of pH-sensitive amphiphilic membrane disruption and endosomal escape leverages the unique physiology of subcellular vesicles to design novel nonlamellar ionizable lipids for safe and efficient cytosolic delivery via systemic administration. Nonamphiphilicity of the LNP at neutral pH results in low hemotoxicity and cytotoxicity, enabling safe systemic and local nucleic acid delivery. Enhanced amphiphilicity of ionizable lipids, triggered by protonation in acidic endosomes, promotes localized destabilization of the endosomal membrane and facilitates the nucleic acid release into the cytosol. Nonlamellar ionizable lipids that are non-amphiphilic at neutral physiological pH but become amphiphilic at early and late endosomal pH show significant pH-sensitive endosomal escape. Nonlamellar amino lipids with small amino head groups and either distant dual unsaturated lipid tails or branched tails that constitute a large wedge-shaped structure exhibit well-controlled pH-sensitive amphiphilicity, enabling them to respond effectively to pH changes during subcellular trafficking post-endocytosis. These lipids have demonstrated high delivery efficiency for various nucleic acids, including mRNA, siRNA, and plasmid DNA, while maintaining safety for systemic *in vivo* applications. The pH-sensitive amphiphilic membrane disruption of ionizable lipids can be characterized by measuring hemolytic activity, which serves as an indicator of their ability to destabilize the lipid bilayer membrane and promote endosomal escape. This mechanism provides a framework for designing novel ionizable lipids and LNPs for safe, efficient, and targeted *in vivo* delivery of nucleic acid therapeutics.
